# Transcriptome Analysis Reveals Anti-Cancer Effects of Isorhapontigenin (ISO) on Highly Invasive Human T24 Bladder Cancer Cells

**DOI:** 10.3390/ijms25031783

**Published:** 2024-02-01

**Authors:** Alex H. Li, Sun Young Park, Peiwei Li, Chaoting Zhou, Thomas Kluz, Jingxia Li, Max Costa, Hong Sun

**Affiliations:** Division of Environmental Medicine, Department of Medicine, NYU Grossman School of Medicine, 341 East 25th Street, New York, NY 10010, USA; ahl421@nyu.edu (A.H.L.); sp3662@nyu.edu (S.Y.P.); peiweili@email.sdu.edu.cn (P.L.); zhouc06@nyu.edu (C.Z.); thomas.kluz@nyulangone.org (T.K.); jingxia.li@nyulangone.org (J.L.); max.costa@nyulangone.org (M.C.)

**Keywords:** bladder cancer, whole transcriptome analysis, tumor microenvironment, actin cytoskeleton, metabolic reprogramming

## Abstract

Bladder cancer, the most common malignancy of the urinary tract, has a poor overall survival rate when the tumor becomes muscle invasive. The discovery and evaluation of new alternative medications targeting high-grade muscle invasive bladder cancer (MIBC) are of tremendous importance in reducing bladder cancer mortality. Isorhapontigenin (ISO), a stilbene derivative from the Chinese herb *Gnetum cleistostachyum*, exhibits a strong anti-cancer effect on MIBCs. Here, we report the whole transcriptome profiling of ISO-treated human bladder cancer T24 cells. A total of 1047 differentially expressed genes (DEGs) were identified, including 596 downregulated and 451 upregulated genes. Functional annotation and pathway analysis revealed that ISO treatment induced massive changes in gene expression associated with cell movement, migration, invasion, metabolism, proliferation, and angiogenesis. Additionally, ISO treatment-activated genes involved in the inflammatory response but repressed genes involved in hypoxia signaling, glycolysis, the actin cytoskeleton, and the tumor microenvironment. In summary, our whole transcriptome analysis demonstrated a shift in metabolism and altered actin cytoskeleton in ISO-treated T24 cells, which subsequently contribute to tumor microenvironment remodeling that suppresses tumor growth and progression.

## 1. Introduction

Bladder cancer is the 10th most prevalent cancer worldwide, with three-quarters of all cases found in men [1,2]. The American Cancer Society estimates that 82,290 new cases of bladder cancer will be diagnosed in 2023, making it the fourth most common cancer in males [3]. Bladder cancer is classified as either non-muscle invasive (NMIBC) or muscle invasive (MIBC). NMIBC has a relatively high overall survival rate, but it often recurs, with approximately 15–20% of cases progressing into the more aggressive MIBC [4]. The current standard treatment for MIBC involves transurethral resection of the bladder tumor (TURBT) and neoadjuvant chemotherapy [5]. Despite recent advances in bladder cancer research, leading to effective targeted treatments and immunotherapies such as PD-L1 inhibitors and FGFR inhibitors, the prognosis for metastatic bladder cancer remains very poor, with only 6% of patients surviving beyond five years post-diagnosis [5,6]. Thus, the development of new therapeutic strategies for bladder cancer treatment is crucial for improving patient outcomes.

Isorhapontigenin (ISO), a stilbene derivative from the Chinese herb *Gnetum cleistostachyum*, has been used for centuries as a therapeutic agent for bronchitis, cardiovascular diseases, and various cancers, including bladder cancer [7]. As an analog of resveratrol [8], ISO displays a similar anti-inflammatory effect in rat chondrocytes through the inhibition of interleukin-1β, inducible nitric oxide synthase (iNOS), prostaglandin E2 (PGE2), and cyclooxygenase-2 (COX-2) [9,10,11]. Additionally, ISO has been shown to protect against oxidative stress in paraquat-induced kidney injury through the overexpression of SOX9 and TOLLIP [12]. Compared to resveratrol, ISO demonstrates enhanced anticancer efficacy and increased bioavailability [13,14,15]. Several studies have reported an inhibitory effect of ISO on bladder tumor growth and progression both in vitro and in vivo [16,17,18,19,20,21]. While low-dose ISO treatment effectively induces G0/G1 growth arrest by suppressing the key cell cycle regulatory protein cyclin D1 expression [22,23], high-dose ISO triggers an apoptotic response of cancer cells by inhibiting anti-apoptotic XIAP gene transcription [7]. In addition, sublethal doses (10–20 µM) of ISO induce cell autophagy via upregulation of SESN2 and BECN1, which further contributes to ISO’s anti-cancer activity [17]. Moreover, in the N-butyl-N-(4-hydroxybutyl) nitrosamine (BBN)-induced mouse bladder cancer model, co-treatment with ISO not only reduces the number of tumors but also limits the tumor from spreading into adjacent muscle tissues, suggesting a strong inhibitory effect of ISO on tumor invasion [19]. Although many molecules and pathways have been proposed to mediate ISO’s anti-cancer activities, a comprehensive analysis of the ISO-altered transcriptome landscape and molecular network has not been previously reported.

In the present study, we conducted a whole transcriptome analysis to identify novel gene targets and signaling pathways by which ISO may act as a therapeutic agent for bladder cancer. Our analysis identified 1047 differentially expressed genes (DEGs) in ISO-treated bladder cancer cells, including 596 downregulated and 451 upregulated genes. Functional annotation and pathway analysis of DEGs revealed that the most important targets of ISO in bladder cancer are the tumor microenvironment and actin cytoskeleton. Additionally, ISO is able to rewire cancer metabolism through inhibiting HIF1α signaling and glycolysis. This comprehensive analysis of the potential mechanisms of action of ISO will provide insight into the evolving landscape of treatments for bladder cancer patients.

## 2. Results

### 2.1. ISO Inhibits T24 Bladder Cancer Cell Viability, Migration, and Invasion

**Figure 1:**
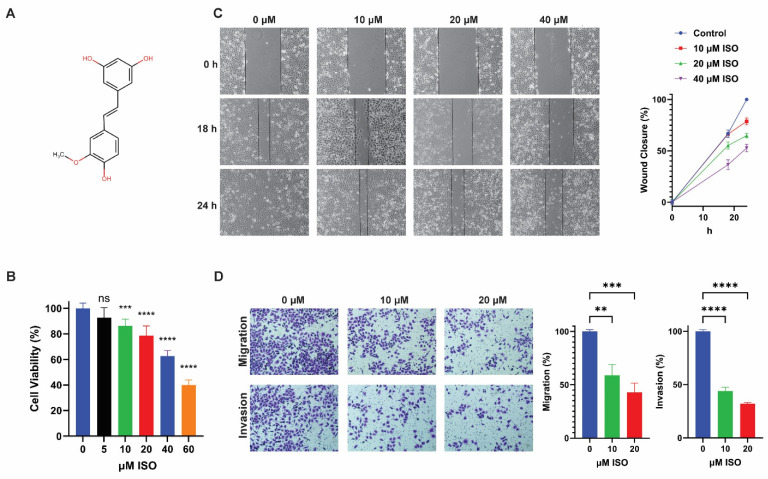
Treatment with ISO decreases cell growth, migration, and invasion in human urinary bladder cancer T24 cells. (**A**) Chemical structure of Isorhapontigenin (ISO). (**B**) MTS assay for cell viability was performed with increasing concentrations of ISO at 24 h (*n* = 8). (**C**) Representative images of in vitro wound healing assays were used to evaluate T24 bladder cancer wound healing rate at 18 and 24 h post-ISO treatment; Line graph illustrates the percentage wound closure at indicated time points during the scratch wound assay. (**D**) T24 cells were subjected to transwell invasion assay in medium containing either vehicle or 20 μM of ISO for 24 h; quantification of migrated cells; quantification of invaded cells. All statistical analyses were performed with one-way ANOVA, followed by Dunnett’s multiple comparison’s tests. ** *p*  <  0.01 *** *p*  <  0.001 **** *p*  <  0.0001, ns, not significant.

ISO is a stilbene derivative with a similar chemical structure to resveratrol (Figure 1A). To examine the effects of ISO on cell viability, we performed the MTS assay in T24 cells treated with varying doses of ISO (0, 5, 10, 20, 40, and 60 μM) for 24 h. ISO treatment reduced cell viability in a dose-dependent manner, as evidenced by a gradual decrease from 92.6% at 5 μM to 40.1% at 60 μM, with significant changes starting from 10 μM compared to the untreated control (Figure 1B). Next, the effects of ISO on cell migration were assessed using the scratch wound healing assay. As shown in Figure 1C, ISO-treated cells displayed delayed wound healing at 24 h, with ISO treatment at 10, 20, and 40 μM doses eliciting 82.4%, 68.6%, and 54.4% wound closure, respectively, compared to full closure (100%) in the untreated control. Moreover, transwell assays confirmed that ISO inhibited both migration and invasion of T24 cells (Figure 1D). When treated with 10 and 20 µM ISO, T24 cell migration was reduced to 58.8% and 42.7%, while cell invasion declined to 43.9% and 32.1%, respectively, compared to those in the untreated controls (Figure 1D). Collectively, these results demonstrated that ISO inhibits human bladder cancer progression via suppressing cancer cell survival, migration, and invasion.

### 2.2. Transcriptome Analysis Reveals Differential Gene Expression in ISO-Treated T24 Cells

**Figure 2:**
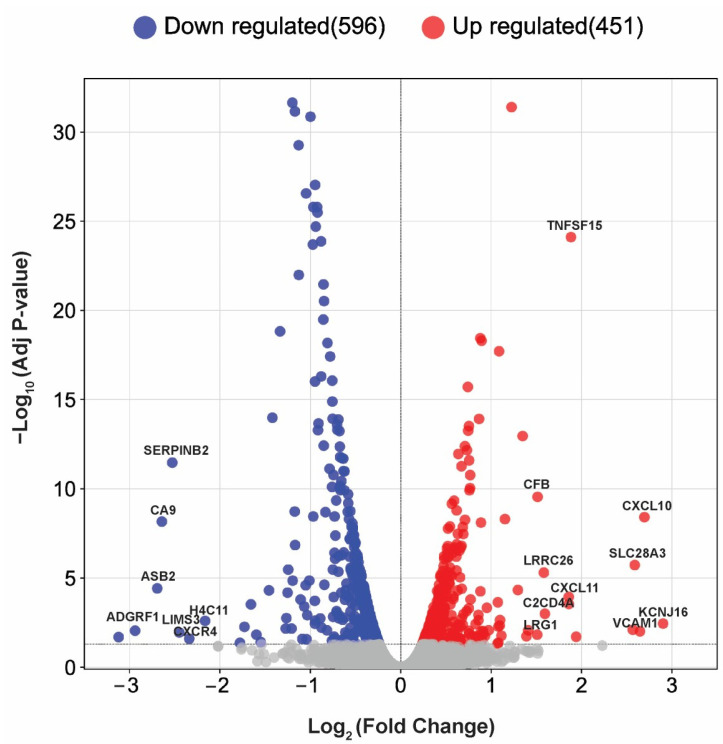
Differential expression of genes in T24 cells treated with or without ISO. Volcano plot of differentially expressed genes (DEGs) in ISO-treated and -untreated T24 cells. Blue dots represent downregulated genes, red dots represent upregulated genes, and grey dots represent insignificant genes. The *X*-axis denotes log2 fold change values and the *Y*-axis shows −log10 adjusted *p*-values.

RNA-sequencing was performed on T24 cells treated with or without 20 µM of ISO for 24 h. A total of 1048 differentially expressed genes (DEGs) were identified between the treatment and control group, of which 596 were downregulated and 451 were upregulated (Figure 2; Supplemental Table S1). The top 20 significantly altered genes, including the 10 most downregulated genes (*KCNK3*, *ADGRF1*, *ASB2*, *CA9*, *SERPINB2*, *LIMS3*, *IGFBP3*, *CXCR4*, *H4C11*, *F3*) and 10 most upregulated genes (*KCNJ16*, *CXCL10*, *CDRT4*, *SLC28A3*, *VCAM1*, *TNFSF15*, *CXCL11*, *C2CD4A*, *LRRC26*, *CFB*, *LRG1*) are listed in Table 1.

**Figure 3:**
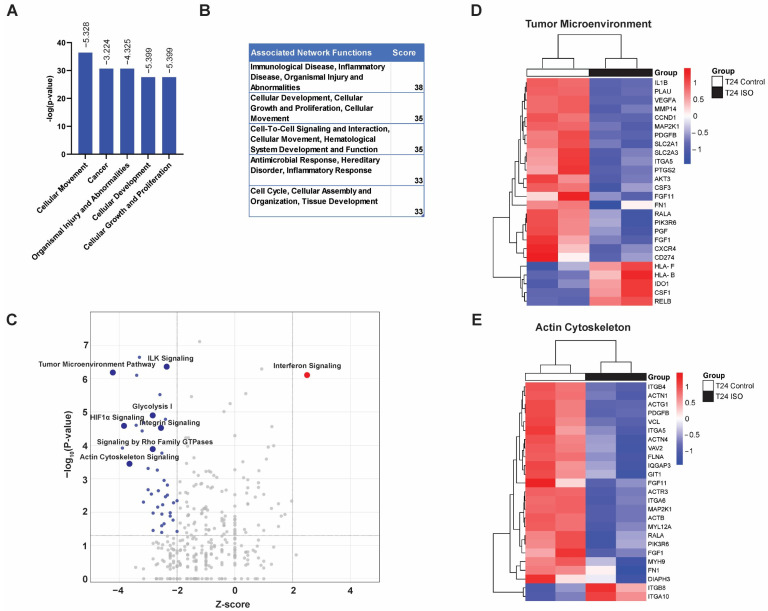
Ingenuity Pathway Analysis (IPA) of top diseases and biological functions, networks, and canonical pathways related to DEGs. (**A**) Bar graph of the −log *p*-values for top diseases and biological functions associated with DEGs. The Z-score of each pathway is displayed above each bar. (**B**) List of top associated network functions associated with DEGs. The score represents the network score. (**C**) Volcano plot of significant canonical pathways enriched in DEGs (*p* < 0.05 and Z-score > 2). Blue dots represent downregulated and red dots represent upregulated canonical pathways. Grey dots are not significant canonical pathways. The *X*-axis depicts the Z-score, and the *Y*-axis shows −log10 *p*-values. (**D**,**E**) Heatmap showing DEG profile of tumor microenvironment (**D**) and actin cytoskeleton (**E**) genes in ISO-treated and control T24 cells.

Ingenuity Pathway Analysis (IPA) was used to determine molecular pathways and cell functions associated with the identified DEGs. The top diseases and biological functions enriched in the DEGs include “Cellular Movement”, “Cancer”, “Organismal Injury and Abnormalities”, “Cellular Development”, and “Cellular Growth and Proliferation” (Figure 3A). The top five associated network functions are “Immunological Disease, Inflammatory Disease, Organismal Injury and Abnormalities”, “Cellular Development, Cellular Growth and Proliferation, Cellular Movement”, “Cell-to-Cell Signaling and Interaction, Cellular Movement, Hematological System Development and Function”, “Antimicrobial Response, Hereditary Disorder, Inflammatory Response”, and “Cell Cycle, Cellular Assembly and Organization, Tissue Development” (Figure 3B). ISO treatment inhibited many canonical pathways, including “Tumor Microenvironment Pathway” (*p*-value = 6.68 × 10^−7^), “HIF1α Signaling” (*p*-value = 6.68 × 10^−5^), and “Actin Cytoskeleton Signaling” (*p*-value = 3.5 × 10^−4^), whereas the only highly activated molecular pathway was “Interferon Signaling” (*p*-value = 7.58 × 10^−7^) (Figure 3C). The expression of DEGs associated with the tumor microenvironment (TME) and actin cytoskeleton were further clustered and visualized using heatmaps (Figure 3D,E). Many TME-related genes (such as *VEGFA*, *MMP14*, *CCND1*, *MAP2K1*, *SLC2A1*) (Figure 3D) and actin cytoskeleton-associated genes (such as *ITGB4*, *ITGA6*, *ACTG1*, *VCL*) (Figure 3E) were downregulated, suggesting a less favorable TME and an altered cytoskeleton network in ISO-treated cancer cells.

Together, these results suggest that ISO treatment induces massive changes in the gene expression of T24 bladder cancer cells, leading to alterations in the tumor microenvironment and actin cytoskeleton, which subsequently contribute to reduced cell survival, migration, and invasion.

### 2.3. Functional Annotation and Pathway Analyses of ISO-Induced DEGs

**Figure 4:**
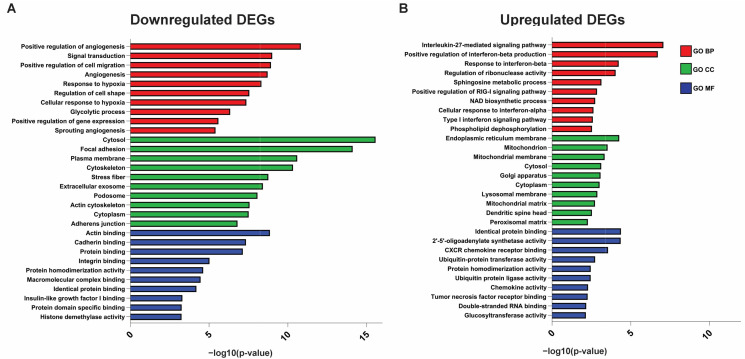
Gene Ontology (GO) enrichment analysis on all DEGs between control and ISO-treated T24 cells. Top enriched biological processes (BPs), cell components (CCs), and molecular functions (MFs) of downregulated (**A**) and upregulated (**B**) DEGs. GO terms were ranked by −log10 (*p*-value).

To further investigate the gene function and molecular pathways altered in ISO-treated cells, Gene Ontology (GO) functional annotation and Kyoto Encyclopedia of Genes and Genomes (KEGG) pathway enrichment were analyzed using the DAVID Annotation. The top biological processes (BP) enriched in ISO-downregulated genes were Positive regulation of angiogenesis (*p*-value = 1.51 × 10^−11^), Signal transduction, (*p*-value = 9.88 × 10^−10^), Positive regulation of cell migration (*p*-value = 1.18 × 10^−9^), Angiogenesis (*p*-value = 1.94 × 10^−9^), Response to hypoxia (*p*-value = 4.84 × 10^−9^), and Regulation of cell shape (*p*-value = 2.77 × 10^−8^) (Figure 4A). Top cellular components (CC) related to downregulated DEGs included Cytosol (*p*-value = 2.70 × 10^−16^), Focal adhesion (*p*-value = 7.67 × 10^−15^), Plasma membrane (*p*-value = 2.54 × 10^−11^), Cytoskeleton (*p*-value = 4.72 × 10^−11^), and Stress fibers (*p*-value = 1.74 × 10^−9^) (Figure 4A). The top five molecular functions (MFs) enriched in ISO-downregulated genes comprised Actin binding (*p*-value = 1.38 × 10^−9^), Cadherin binding (*p*-value = 4.52 × 10^−8^), Protein binding (*p*-value = 7.16 × 10^−8^), Integrin binding (*p*-value = 9.38 × 10^−8^), and Protein homodimerization activity (*p*-value = 2.38 × 10^−7^) (Figure 4A). Together, functional annotation of downregulated genes suggested that ISO suppressed the expression of genes related to the actin cytoskeleton, focal adhesions, cadherin binding, and integrin binding, which may lead to compromised cell movement. For ISO-upregulated genes, the top biological processes enriched were Interleukin-27-mediated signaling pathway (*p*-value = 8.59 × 10^−8^), Positive regulation of interferon-beta production (*p*-value = 1.95 × 10^−7^), Response to interferon-beta (*p*-value = 5.90 × 10^−5^), Regulation of ribonuclease activity (*p*-value = 9.24 × 10^−5^), and Sphingosine metabolic process (*p*-value = 7.28 × 10^−4^) (Figure 4B). The highly enriched cellular components included the Endoplasmic reticulum membrane (*p*-value = 5.74 × 10^−5^), Mitochondrion (*p*-value = 3.01 × 10^−4^), Mitochondrial membrane (*p*-value = 4.55 × 10^−4^), Cytosol (*p*-value = 7.54 × 10^−4^), and Golgi apparatus (*p*-value = 8.29 × 10^−4^) (Figure 4B). The top molecular functions in upregulated genes were Identical protein binding (*p*-value = 4.25 × 10^−5^), 2′-5′-oligoadenylate synthetase activity (*p*-value = 4.44 × 10^−5^), CXCR chemokine receptor binding (*p*-value = 2.82 × 10^−4^), Ubiquitin-protein transferase activity (*p*-value = 0.00184), and Protein homodimerization activity (*p*-value = 0.00348) (Figure 4B). Together, GO functional annotation analysis suggested that ISO treatment activated genes involved in the inflammatory pathway but repressed genes related to hypoxia signaling, glycolysis, and angiogenesis, leading to an altered tumor microenvironment that suppresses tumor growth and progression.

**Figure 5:**
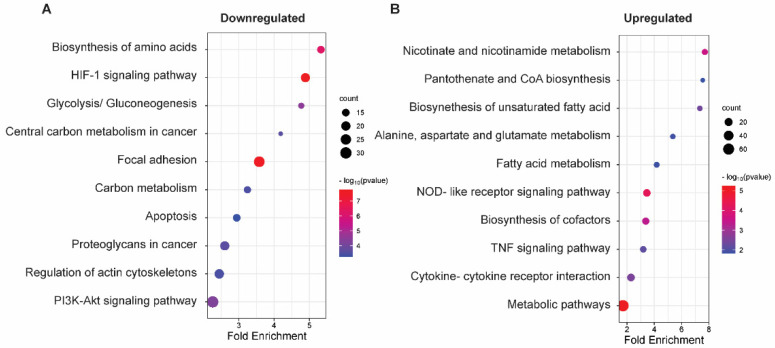
Kyoto Encyclopedia of Genes and Genomes (KEGG) pathway enrichment analysis of DEGs. KEGG pathway enrichment analysis of downregulated (**A**) and upregulated (**B**) DEGs. The *Y*-axis indicates KEGG pathways, and the *X*-axis indicates fold enrichment of the pathways. Bubble color and size correspond to −log (*p*-value) and the number of genes enriched in the pathway, respectively.

Top KEGG pathways that were enriched in ISO-downregulated genes were Biosynthesis of amino acids (*p*-value = 5.59 × 10^−7^), HIF-1 signaling pathway (*p*-value = 1.67 × 10^−8^), Glycolysis/Gluconeogenesis (*p*-value = 3.32 × 10^−5^), Central carbon metabolism in cancer (*p*-value = 2.51 × 10^−4^), Focal adhesion (*p*-value = 2.60 × 10^−8^), Regulation of actin cytoskeletons (*p*-value = 3.51 × 10^−4^), and PI3K-Akt signaling pathway (Figure 5A). In upregulated genes, top KEGG pathways included Nicotinate and nicotinamide metabolism (*p*-value = 2.42 × 10^−4^), Pantothenate and CoA biosynthesis (*p*-value = 0.015), Biosynthesis of unsaturated fatty acid (*p*-value = 0.0043), Alanine, aspartate, and glutamate metabolism (*p*-value = 0.013), and Fatty acid metabolism (*p*-value = 0.014) (Figure 5B). Other cancer-related KEGG terms such as Metabolic pathways (*p*-value = 5.90 × 10^−6^), Cytokine–cytokine receptor interaction (*p*-value = 3.0 × 10^−3^), and TNF signaling pathway (*p*-value = 7.03 × 10^−3^) were also enriched in ISO-upregulated genes, which was consistent with our GO analysis (Figure 4B and Figure 5B). Thus, KEGG pathway analyses demonstrated that ISO targets the metabolic reprogramming of bladder cancer and induces substantial changes in major metabolic pathways, which subsequently impedes cancer cell survival, migration, and invasion.

### 2.4. Gene Set Enrichment Analysis (GSEA)

**Figure 6:**
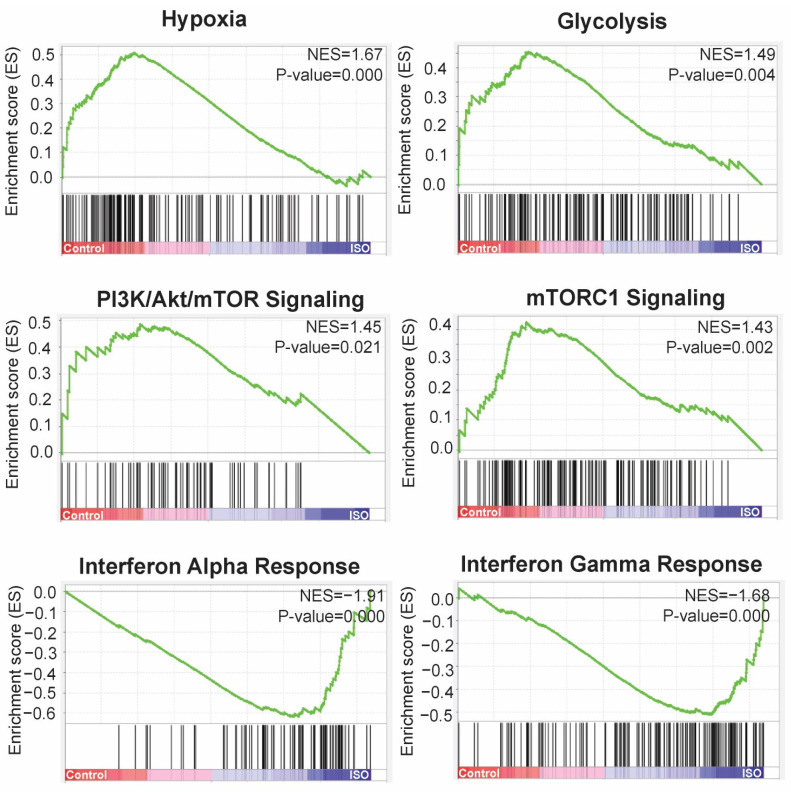
Gene set enrichment analysis (GSEA) of ISO-treated T24 cells. GSEA enrichment plots were created using the most negatively enriched and positively enriched gene sets in ISO-treated T24 cells. The enrichment score reflects the maximum deviation from the random distribution. Positive enrichment scores (ESs) indicate gene set enrichment at the top of the ranked list; negative ES indicate gene set enrichment at the bottom of the ranked list.

Gene set enrichment analysis (GSEA) was performed to analyze the coordinated changes of the Hallmark gene set (MsigDB, Broad Institute) [24] between ISO-treated and untreated control samples. Results showed that 6 out of 50 hallmarks were significantly enriched (Figure 6). Hypoxia (NES = 1.67, FDR q = 0.030, *p* = 0.000), Glycolysis (NES = 1.49, FDR q = 0.121, *p* = 0.004), PI3K/Akt mTOR Signaling (NES = 1.45, FDR q = 0.139, *p* = 0.021), and mTORC1 Signaling (NES = 1.43, FDR q = 0.119, *p* = 0.002) were highly enriched in untreated control samples, whereas Interferon Alpha Response (NES = −1.91, FDR q = 0.001, *p* = 0.000) and Interferon Gamma Response (NES = −1.68, FDR q = 0.014, *p* = 0.000) were robustly enriched in ISO-treated samples (Figure 6). Consistent with the findings from the functional annotation and pathway analysis, the GSEA analysis indicated that ISO treatment, compared to the untreated controls, induced the expression of genes related to the inflammatory response and suppressed those involved in metabolic reprogramming.

### 2.5. Validation of Downregulated Genes upon ISO Treatment in T24 Cells

**Figure 7:**
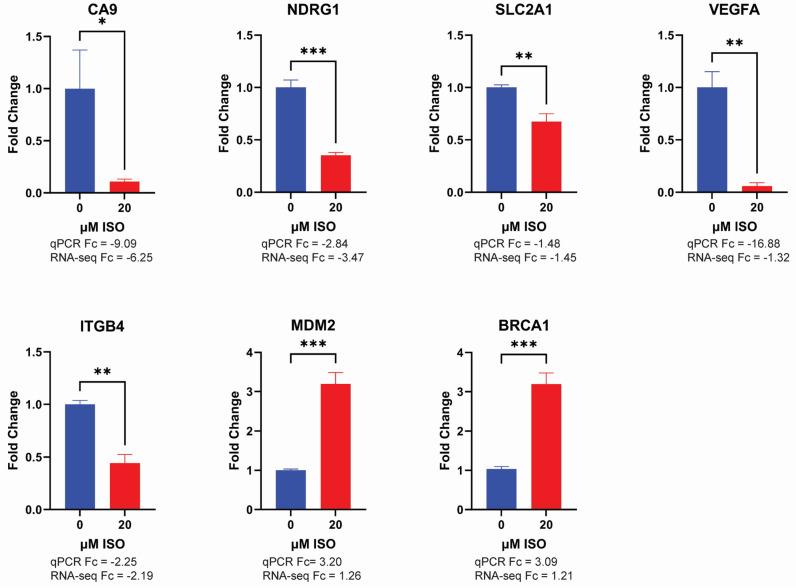
Validation of gene expression by quantitative real-time PCR. Relative mRNA expression levels of *CA9*, *NDRG1*, *SLC2A1*, *VEGFA*, *ITGB4*, *MDM2*, and *BRCA1* were analyzed in T24 cells treated with ISO for 24 h or untreated control cells. Gene expression was normalized relative to the expression of β-Actin and presented as fold change to the levels of untreated control cells. Fold changes of each gene from qPCR and RNA-seq were listed. Data are presented as mean ± SD (*n* = 3). Significance was determined using an unpaired *t*-test. * *p*  <  0.05, ** *p*  <  0.01, *** *p*  <  0.001.

Seven genes, including five downregulated genes (*CA9*, *ITGB4*, *NDRG1*, *SLC2A1*, and *VEGFA*) and two upregulated genes (*MDM2*, and *BRCA1*), were chosen for validation with RT-qPCR. The *CA9* gene encodes a protein responsible for cellular adaptation to acidosis by facilitating the hydration of carbon dioxide into bicarbonate ions and protons [25]. Integrin β4 (ITGB4) is a transmembrane receptor critical for epithelial–mesenchymal transition (EMT) and actin cytoskeleton organization [26]. Increased ITGB4 expression is often associated with cancer invasiveness and angiogenesis [27]. N-myc downstream regulated 1 (NDRG1), a protein commonly upregulated in various cancers, including bladder cancer, is implicated in stress responses, cell growth, and differentiation [28]. GLUT1 (SLC2A1) is a glucose transporter protein highly associated with bladder cancer malignancy [29]. As an important regulator for metabolic flux, reduced SLC2A1 expression suggests suppression of glycolysis and the metabolic programming [30]. Vascular endothelial growth factor (VEGFA), a key protein in tumor angiogenesis [31], is a critical downstream target of HIF1α signaling and plays an important role in tumor invasion and metastasis [32]. Mouse double minute 2 homolog (MDM2), an E3 ubiquitin-protein ligase, is a negative regulator of p53 and promotes cell growth, angiogenesis, metabolic reprogramming, metastasis, and immune suppression [33]. Breast cancer type 1 susceptibility protein (BRCA1), a tumor suppressor gene commonly mutated in breast and ovarian cancer, plays a critical role in maintaining genomic integrity [34]. As shown in Figure 7, RT-qPCR analysis revealed that ISO downregulates CA9, NDRG1, SLC2A1, VEGFA, ITGB4, and upregulates MDM2 and BRCA1 in T24 cells, which is consistent with RNA-seq results. Moreover, downregulation of *CA9*, *NDRG1*, *SLC2A1*, *VEGFA*, and upregulation of *MDM2* were further verified in an additional bladder cancer cell line, 5637 cells (Supplemental Figure S1).

## 3. Discussion

ISO, a stilbene derivative from Chinese herbs, displays a strong inhibitory effect on muscle invasiveness and progression of human MIBCs, yet the mechanism underlying this inhibitory effect remains unclear. In the present study, we conducted the first whole transcriptome profiling of ISO-treated bladder cancer cells to identify key molecules and pathways involved. A total of 1047 differentially expressed genes were identified and subjected to functional annotation and pathway analysis. Our results showed that ISO treatment of MIBCs suppresses genes associated with the actin cytoskeleton, HIF1α signaling, and cell metabolism, while enhancing the transcription of genes associated with interferon signaling. Further analysis suggests that ISO-induced transcriptome changes resulted in a shifted tumor microenvironment to suppress cell growth, survival, migration, and invasion.

Previous studies have demonstrated a strong inhibitory effect of ISO in bladder cancer initiation and progression [7,19,20,22,35,36,37]. ISO treatment at 5–10 µM inhibits cell proliferation and induces cell cycle G0/G1 arrest in human bladder cancer cells [22]. Cyclin D1 protein was significantly downregulated by ISO in UMUC3, RT112, and RT4 cells. Ectopic expression of Cyclin D1 in UMUC3 cells reversed ISO-induced G0/G1 growth arrest, suggesting that the downregulation of Cyclin D1 by ISO contributes to its cancer inhibitory effect. Similarly, our results showed that T24 cells treated with 20 µM ISO exhibited a significant reduction in cyclin D1 (*CCND1*) expression, further supporting cyclin D1 as a crucial target mediating ISO’s anticancer activity. In addition, a high dose of ISO (20–60 µM) induced apoptosis in T24T, UMUC3, and RT112 cells by suppressing XIAP, a ring domain-containing anti-apoptotic protein [7]. Even though XIAP was not among the DEG list, the Ingenuity Pathway Analysis of DEGs indicated a significant enrichment of “cell death of tumor cell lines” significantly enriched in ISO-treated T24 cells (z-score = +2.203, *p*-value = 4.62 × 10^−32^). Consistently, the MTS assay demonstrated a dose-dependent reduction in cell viability upon ISO treatment (Figure 1B). Moreover, several studies have reported that ISO inhibits cell migration and invasion in T24T, 5637, and UMUC3 cells by targeting various pathways, including the STAT1/FOXO1 axis, GSK3b-HSP70-MMP-2 axis, and several EMT markers such as vimentin in T24T and 5637 cells [16,18,19,20,21]. Similarly, our study revealed a similar inhibitory effect of ISO on T24 cell migration and invasion (Figure 3C,D). Interestingly, our RNA-seq analysis did not encompass some individual gene changes reported in previous studies, probably due to the use of different cell lines. It is worth noting that our study primarily investigates T24 cells, while others used UMUC3, T24T, and 5637 cells. It is worth noting that our study revealed a similar inhibitory effect of ISO on T24 cell migration and invasion (Figure 3C,D). Using multiple functional annotation approaches, our analysis revealed that ISO treatment significantly influenced cell movement, likely through the reorganization of the actin cytoskeleton, altered cell adhesion, and downregulation of Rho-GTPase signaling (as discussed below). Taken together, our analysis, in concert with numerous previous reports, strongly supports the anticancer activity of ISO in bladder cancer.

The reorganization of the actin cy”oske’eton Is a prerequisite for cancer cell migration and invasion [38]. Our study demonstrated that 23 out of 25 DEGs associated with the actin cytoskeleton pathways were downregulated with ISO (Figure 3E). ISO treatment significantly suppressed genes encoding various actin proteins (*ACTG1*, *ACTN1*, *ACTN4*, *ACTB*, *ACTR3*). As key structural components in the cytoskeleton system, actin proteins play important roles in many cellular processes, including cell division and migration [39,40,41]. Aberrant expression of actin isoforms has been reported in many human cancers and serves as the biomarker for tumor initiation and progression [40,42]. For example, dysregulation of *ACTG1* has been reported in lung cancer, colorectal cancer, prostate cancer, and breast cancer [43,44,45,46]. Downregulation of *ACTG1* in prostate cancer cells inhibited cell proliferation, migration, and invasion [43]. Similarly, a potential mechanism by which ISO inhibits bladder cancer cell proliferation, migration, and invasion may be through downregulation of ACTG1 (Figure 3E). In addition to actin proteins, other components of the pathway are also downregulated by ISO, including myosin heavy and light chains (*MYH9*, *MYL12A*), fibronectin 1 (*FN1*), and several integrin receptors (*ITGB4*, *ITGA5*, *ITGA6*) (Figure 3E). Integrin receptors link the extracellular matrix environment to the actin cytoskeleton and are critical components in focal adhesions [47,48]. Increased expression of *ITGB4* and *ITGA5* are associated with poor cancer prognosis with increased cancer migration and invasion [49,50,51,52]. Our analysis revealed that *ITGB4/ITGA6* and *ITGA5* were significantly downregulated with ISO treatment, suggesting that ISO may suppress cell migration and invasion of bladder cancer cells by decreasing integrin expression and activities (Figure 3E). It is worth noting that several members of the Rho-GTPases family are also downregulated by ISO, including *RHOB*, *RHOC*, *RHOF*, and *RHOU* (Supplemental Table S1). Rho GTPases, the small GTPases of the Ras superfamily, play important roles in cytoskeletal dynamics and cell motility [53]. The downregulation of Rho-GTPase signaling suppresses actin cytoskeleton remodeling, consequently inhibiting cancer cell migration and invasion [41]. Taken together, ISO treatment suppressed the expression of genes related to integrin signaling and actin cytoskeleton remodeling, leading to reduced cell migration and invasion.

Hypoxia or HIF1α signaling has been identified as another pathway suppressed significantly by ISO across various pathway analysis platforms, such as IPA canonical pathway analysis (Figure 3), GO function enrichment (Figure 4), KEGG pathway analysis (Figure 5), and GSEA analysis (Figure 6). HIF1α is a key regulator in the adaptation of cancer cells to hypoxic conditions commonly found in tumor microenvironments and plays important roles in metabolic reprogramming, cell migration, stem cell renewal, and angiogenesis [54,55,56]. Elevated HIF1α protein levels have been reported in numerous human cancers and are closely linked with tumor malignancy [57]. ISO treatment significantly suppressed a large number of HIF1α downstream targets, including genes involved in cell growth and survival (*NDRG1*, *ADM*, *IGF2BP2*, *IGFBP3*), migration and invasion (*CXCR4*, *VEGFA*, *MMP14*), angiogenesis (*HMOX1*, *ADM*, *VEGFA*), as well as those related to glucose metabolism (*SLC2A1*, *HK2*, *PGK1*) (Supplemental Table S1). Metabolic reprogramming is a hallmark of human cancers and is essential for both malignant transformation and tumor development [58,59]. In solid tumors, elevated HIF1α orchestrates a metabolic shift from oxidative phosphorylation to glycolysis in order to supply the necessary energy to support cancer cell growth and function [54]. There has been an increasing focus in cancer therapeutics on manipulating HIF1α signaling to reconfigure cancer metabolism, which has been a growing interest in cancer therapeutics [60,61]. Consistent with the suppression of HIFα, ISO treatment significantly inhibited glycolysis (Figure 3C, 4A, 5A, and 6) through the suppression of genes such as *AKT3*, *SLC2A1*, *PFKP*, *HK1*, *HK2*, *SLC1A5*, *SLC7A5*, *MAP2K1*, *PDK1*, *LDHA*, and *PGAM1* (Supplemental Table S1). *SLC2A1* encodes GLUT1, a glucose transporter commonly upregulated in many forms of cancer, including bladder cancer [30,62]. Through the suppression of *SLC2A1* transcription, ISO targets the uptake of glucose that is critical for maintaining the increased glycolytic flux [30]. HK1 and HK2, two hexokinases acting as the first-rate limiting step of glycolysis [63], were transcriptionally repressed in ISO-treated T24 cells. Depletion of HK2 abolished the initiation and progression of lung cancer and breast cancer both in vitro and in vivo [64]. The transcriptional repression of *PDK1* (pyruvate dehydrogenase kinase 1) upon ISO treatment enables the conversion of pyruvate to acetyl-CoA by pyruvate dehydrogenase (PDH), thereby enabling a shift away from glycolysis [65]. This effect is compounded by the suppression of LDHA, a critical glycolytic enzyme responsible for lactate production [66]. Our results demonstrated a significant inhibitory effect of ISO on genes involved in HIF1α signaling and the glycolysis pathway, suggesting that ISO treatment may reverse the metabolic reprogramming in cancer cells and lead to impaired tumor growth and progression.

Our GSEA analysis revealed that the PI3K/AKT/mTOR pathway and the mTORC1 pathway were significantly inhibited by ISO treatment (Figure 6). Closely related to the HIF1α signaling and glycolysis pathway, the PI3K/Akt/mTOR pathway plays critical roles in cell growth, survival, migration, and metabolism [67,68]. Aberrant activation of the pathway has been reported in many human cancers, contributing to both tumor initiation and progression [69,70,71]. In bladder cancer, this pathway is hyperactivated and contributes to the metabolic reprogramming from oxidative phosphorylation to glycolysis, partly through increased HIF1α [30,72]. PI3K consists of the catalytic p110 subunit and the regulatory subunit, p85 (class IA) or p87 (class IB), which mediate PI3K activation by growth factor receptor tyrosine kinases (RTKs) or G protein coupled receptors (GPCRs), respectively [67,70,73]. PI3K phosphorylates, PIP2 to PIP3, initiate the activation and recruitment of pleckstrin homology (PH) domain-containing proteins, including PI3k-dependent kinase-1 (PDPK1) and its substrate AKT. Downstream of Akt is mTOR, a serine/threonine kinase that is a part of two complexes, mTORC1 and mTORC2 [74]. While mTORC1 activates transcription factors, such as ATF4, SREBP, and HIF1α, leading to altered metabolism and increased cell growth and survival, mTORC2 signaling regulates cytoskeletal remodeling, glucose metabolism, and apoptosis [68]. In ISO-treated T24 cells, several genes involved in the PI3K/AKT/mTOR pathway were significantly downregulated, including *PIK3R6*, *AKT3*, *RPTOR*, *RRAGD* (Supplemental Table S1). *PIK3R6* encodes a regulatory subunit p87PIKAP that binds to a catalytic subunit p110γ to form Class IB PI3K heterodimers [67,75]. P87PIKAP/p110gamma interaction is critical for Ras-mediated G protein-coupled receptor-induced activation of PI3K signaling [76]. RPTOR, the regulatory protein associated with mTOR, is a core component of the mTORC1 complex and facilitates the recruitment of canonical mTORC1 substrates [68]. Upregulation of *RAPTOR* has been reported in colorectal cancer (CRC) and renal cancer cells, promoting CRC cell proliferation and contributing to resistance to the PI3K-mTOR inhibitor [77,78]. Four single nucleotide polymorphisms (SNPs) in the *RAPTOR* gene were significantly associated with increased risk of bladder cancer [79]. RRAGD, a subunit of Rag GTPase heterodimers, plays an important role in mTORC1 activation [80,81]. Elevated *RRAGD* expression has been reported in human hepatocellular carcinoma [82]. Transcriptional activation of *RRAGD* promotes cancer growth [83]. Concurrent with the inhibition of the PI3K/AKT/mTOR pathway, ISO-upregulated *PIK3IP1* is a negative regulator of the PI3K/AKT pathway (Supplemental Table S1). Additionally, mTORC2 is able to phosphorylate and activate Akt, resulting in a positive feedback loop within the PI3K/Akt/mTOR pathway [84]. Akt activation promotes cell survival through phosphorylating and inactivating FOXO transcription factor proteins, thereby impeding transcription of the CDK inhibitor proteins, p21 and p27 [85]. In concert with downregulated PI3K/Akt/mTOR signaling, ISO has been previously reported to increase the expression and activity of FOXO1 and FOXO3a in bladder cancer cells [18,19,37]. Furthermore, ISO-induced upregulation of p27 (*CDKN1B*) and p21 (*CDKN1A*) and downregulation of cyclin D1 (*CCND1*), one of the downstream targets of mTORC1 signaling, were found in our study as well as previous studies [22,37,86,87]. Taken together, ISO suppresses the PI3K/AKT/mTOR pathway in bladder cancer cells and by extension, targets cancer cell metabolism, survival, and growth.

Our analysis showed that the only pathway to be upregulated upon ISO treatment in T24 cells was interferon signaling (Figure 3, Figure 4 and Figure 6). Interferon signaling plays a key role in the activation of both innate and adaptive immune responses through three major types of interferons [88,89]. While type I interferons (IFNα, IFNβ, IFNϵ, IFNκ, IFNω) and type III interferons (IFNλ) activate the STAT1-STAT2 complex, type II interferon (IFNγ) and some type I interferons activate STAT1 homodimers [90]. Subsequently, STAT1 homodimers or STAT1-STAT2 heterodimers translocate into the nucleus and bind to GAS (IFNγ-activated sites) or ISR (IFN-stimulated response) elements to initiate transcription of downstream target genes [91]. Our results showed that genes involved in interferon signaling were upregulated by ISO, including *STAT1*, *IRF9* (an IFN target protein that binds and facilitates the activity of STAT1/STA2 heterodimers), and many downstream target genes (*IFIT1*, *IFIT3*, *IFI6*, *IFI35*, *IFITM1*), suggesting the activation of both IFNα (type I) and IFNγ (type II) signaling (Supplemental Table S1). IFNα, the first immunotherapeutic drug approved by the FDA for cancer treatment, displayed multiple anti-tumor activities [92,93,94]. IFNα not only restricted angiogenesis and metastasis but also stimulated the cytotoxic activity of macrophages, NK cells, and dendritic cells [93]. IFNγ, the only member of type II interferon, is a pleiotropic cytokine that activates cell immunity and stimulates antitumor immune response [95]. IFNγ can be detected in the urine of bladder cancer patients following intravesical Bacillus Calmette–Guerin therapy [96]. It facilitates tumor growth inhibition after immune checkpoint blockade [97]. Consistent with upregulated interferon signaling, CXCL10 and CXCL11, two CXCR3 ligands highly induced by IFN signaling, were increased in T24 cells following ISO treatment [93,95]. To conclude, ISO treatment was found to suppress type I and type II interferon signaling, thereby suppressing tumor growth, angiogenesis, and metastasis.

The tumor microenvironment (TME) is a dynamic complex where cancer cells create tumor-supportive conditions by reprogramming non-cancer cells and remodeling the extracellular matrix (ECM) [98,99]. In addition to tumor cells, the TME comprises immune cells (such as T-cells, B-cells, natural killer cells, dendritic cells, tumor-associated macrophages, and tumor-associated neutrophils) and stromal cells (including tumor-associated fibroblasts, mesenchymal stem cells, tumor endothelial cells, tumor-associated adipocytes, and pericytes) [100,101]. The communication between cancer cells and their surrounding cells, either through direct cell-to-cell interaction or indirect paracrine signaling involving cytokines, chemokines, growth factors, and extracellular vesicles, is essential for creating a tumor-promoting environment and serves as an important target for cancer therapy [98,99,102]. Among the genes involved in the TME, 21 were found to be downregulated by ISO treatment, including several growth factors, *FGF1*, *PGF*, *PDGFB*, *FGF11*, and *VEGFA*. Overexpression of fibroblast growth factor (FGF) family proteins, such as FGF1 and FGF11, is associated with tumor growth, progression, invasion, and metastasis [103]. Overexpression of placental growth factor (PGF) and VEGFA, as members of the VEGF family proteins, correlates with tumor growth and progression in various cancers [104,105,106]. Platelet derived growth factor (PDGFB) overexpression is observed in many cancers, contributing to cell proliferation, angiogenesis, migration, and invasion [107]. The downregulation of these growth factors by ISO inhibits not only tumor growth but also the stromal cell function, such as angiogenesis and EMC remodeling. Furthermore, ISO treatment stimulates the expression of several chemokines, including *CXCL2*, *CXCL3*, *CXCL4*, *CXCL6*, *CXCL10*, *CXCL11,* and *CCL5.* These chemokines can recruit anti-tumor immune cells [108]. For instance, chemokine ligands CXCL10 and CXCL11 can bind to the receptor CXCR3, guiding the migration of T cells and NK cells into solid tumors [108]. CXCL2 and CXCL6 can bind to CXCR1/CXCR2 and recruit neutrophiles [108]. CXCL4, CXCL10, and CXCL11 are known to inhibit angiogenesis and cell proliferation, thus targeting the angiogenic nature of the tumor microenvironment [109]. Therefore, the transcriptome changes induced by ISO may modulate cell-to-cell communication between bladder cancer cells and stromal cells, leading to reduced cell proliferation, invasion, and angiogenesis, while targeting the communication between bladder cancer cells and immune cells to foster an immunosuppressive environment.

In conclusion, we conducted the first transcriptome profiling on human MIBC cells treated with ISO, a natural compound displaying multiple anti-cancer activities. Our results demonstrated that the source of ISO’s multiple anticancer activities lies in its ability to modulate actin cytoskeleton signaling, rewire tumor metabolic reprogramming, and activate interferon signaling, thereby remodeling the tumor microenvironment and inhibiting cancer cell survival, proliferation, migration, and invasion. Further investigation of how ISO targets key molecules and pathways such as HIF1a signaling and the PI3K/AKT/mTOR pathway to facilitate its cancer inhibitory effects, is necessary for maximizing its effectiveness as a cancer therapeutic drug.

## 4. Materials and Methods

### 4.1. Cell Culture

T24 cells, representing high-grade and invasive transitional bladder carcinoma cells, were established from a female patient diagnosed with grade III urinary bladder carcinoma [110]. 5637 cells were isolated from a male patient with grade II carcinoma. T24 (HTB-4) and 5637 (HTB-9) cells were obtained from the American Type Culture Collection (ATCC). The cells were cultured in Dulbecco’s Modified Eagle Medium/Nutrient Mixture F-12 (DMEM/F12; Gibco, Billings, MT, USA) medium supplemented with 5% fetal bovine serum (FBS) and 1% penicillin/streptomycin at 37 °C and 5% CO_2_. ISO (Sigma–Aldrich Corporation, Burlington, MA, USA) with over 98% purity was dissolved in dimethyl sulfoxide (Sigma–Aldrich Corporation).

### 4.2. Cell Viability Assay

T24 cells were seeded at 4000 cells/well in a 96-well plate and treated with various concentrations of ISO (0, 5, 10, 20, 40, 60 μM) for 22 h. Cell viability was determined using CellTiter 96^®^ Aqueous One Solution Cell Proliferation Assay (MTS; Promega; Madison, WI, USA) with the Spectramax M2e Multimode Microplate Reader (Molecular Devices, Winnersh, UK). Cell viability was evaluated based on absorbance and expressed as a percentage of the untreated control.

### 4.3. Wound Healing Assay

T24 cells were seeded in 6-well plates and cultured to 90% confluency. A wound gap was created by scratching with a 20 µL pipette tip. Cells were rinsed with phosphate-buffered saline (PBS; Gibco) several times to remove debris before replacing with fresh media and incubating in a 37 °C incubator. Cells were imaged using a Nikon TS100 microscope (Nikon, Tokyo, Japan) at 0, 18, and 24 h after the initial scratch. Three random fields were marked and measured at each timepoint using ImageJ (Version 1.53t). Wound closure was evaluated by measuring the distance of wound closed and presented as a percentage of the width of the initial wound. Wound closure is presented as the percentage of the total distance of wound closed compared to the distance of the initial wound.

### 4.4. Transwell Invasion Assay

To conduct the transwell invasion assay, 1 × 10^4^ T24 cells were suspended in 200 μL DMEM/F12 with 0.1% FBS in Falcon^®^ Permeable Support in 24-well plates with 8.0 µm of Transparent PET Membrane (Corning, Corning, NY, USA), precoated with 200 μg/μL of Matrigel (Corning). Transwell inserts were placed in 24-well plates containing 600 μL of the completed medium to act as a chemoattractant in the lower chamber. DMEM/F12 in both chambers were supplemented with various concentrations of ISO (0, 10, or 20 μM). After incubation for 48 h in a 37 °C incubator, the chambers were briefly washed with PBS prior to being fixed with 3.7% formalin, and then methanol, before staining with 0.1% crystal violet in PBS. Cells that remained in the top chamber were scraped off with cotton swabs. Stained cells were imaged using an inverted microscope. Quantification of the number of migrated and invaded cells was performed on ImageJ, using three random fields per well. The results are illustrated as bar graphs depicting the mean number of cells counted in each field (*n* = 3), created using GraphPad Prism 10.

### 4.5. RNA Isolation and Sequencing Library Preparation

T24 cells were treated with 20 µM ISO for 24 h. DMSO was added as a vehicle control. Total RNA was extracted from control and ISO-treated T24 cells (*n* = 2) using the TRI-zol reagent (Life Technologies, Gaithersburg, MD, USA). RNA-Seq libraries were prepared using the Illumina TruSeq RNA-sample preparation kit according to the manufacturer’s instructions. Sequencing was performed at the NYU School of Medicine Genome Technology Center using Illumina HiSeq. 2500 by multiplexed paired-end run with 50 cycles.

### 4.6. RNA Sequencing Data Analysis

Raw sequence data (Fastq) were loaded into Biomedical Genomics Workbench Version 22.0.2 (Qiagen, Germantown, MD, USA) for data analysis. The raw Fastq files were trimmed to remove any remaining adaptors and ambiguous nucleotides. The trimmed sequence files were aligned to the human genome (Hg38) allowing two mismatches. Reads mapped to the exons of a gene were summed at the gene level. Gene expression levels were quantified as reads per kilobase of transcript per million reads mapped (RPKM). Differential gene expression was analyzed using the Advance RNA-seq plug-in tool (Qiagen) by comparing each treated group versus the corresponding control group. TMM (trimmed mean of M values) normalization was performed to adjust library sizes before differential expression analysis. The genes with a false discovery rate (FDR)  <  0.05 between the control and treated group, as well as an average mean of total counts no less than 10, were defined as differentially expressed genes (DEGs).

### 4.7. Gene Network, Functional Enrichment, and Pathway Analysis

Gene network, top canonical pathways, and disease and function were analyzed using Ingenuity Pathway Analysis (IPA, Ingenuity^®^ Systems, www.ingenuity.com (accessed on 2 April 2023)). Functional enrichment analysis was assessed using the Database for Annotation, Visualization, and Integrated Discovery (DAVID) database (http://david.ncifcrf.gov/www.ingenuity.com (accessed on 2 April 2023)), an NCBI web-based functional annotation tool. Gene Ontology (GO) analysis results were categorized under “biological processes” (BP), “cellular component” (CC), and molecular function (MF). Kyoto Encyclopedia of Genes and Genomes (KEGG) pathway enrichment analysis was conducted via DAVID to identify significantly enriched signal transduction pathways. Volcano plots of genes and canonical pathways and bubble-plots of the KEGG pathway analysis were created using SRplot. Graphs illustrating GO pathway analysis and changes in disease and function were created using Prism GraphPad 10.

### 4.8. Gene Set Enrichment Analysis (GSEA)

Gene set analysis was analyzed by GSEA software (Version 4.3.2, http://software.broadinstitute.org/gsea/index.jsp (accessed on 29 July 2023)) using the Hallmark gene set collection (MSigDB v7.0, Broad Institute, Cambridge, MA, USA). The enrichment score for each gene set is calculated using the entire ranked list, which reflects how the genes for each set are distributed in the ranked list. Normalized enriched score (NES) was determined for each gene set. The significant enrichment of gene set was selected based on the absolute values of NES > 1, nominal *p*-value of NES ≤ 0.05, and false discovery rate (FDR) ≤ 0.05.

### 4.9. Quantitative Reverse Transcription Polymerase Chain Reaction (RT-qPCR)

Total RNA was extracted with TRIzol (Invitrogen, Waltham, MA, USA) from T24 cells or 5637 cells treated with 20 μM of ISO for 24 h according to the manufacturer’s instructions. RNA concentrations were determined using a Nanodrop 2000. cDNA was synthesized using LunaScript RT Master Mix (New England Biolabs, Ipswich, MA, USA) according to the manufacturer’s protocol, using a Biorad T100 thermocycler (Biorad). qPCR was performed using PowerUP SYBR Green Supermix (Thermofisher, Waltham, MA, USA) using the QuantStudio 6 (Invitrogen). The primers used are listed in Supplemental Table S2. The relative gene expression was analyzed by the $2^{-\Delta \Delta Ct}$ method [111], and all values were normalized to β-actin.

### 4.10. Statistical Analysis

GraphPad Prism10 software was used to conduct statistical analysis. To determine significance, one-way ANOVA with Dunnett’s multiple comparisons test was applied to transwell migration and invasion and MTS assays. Significance for relative mRNA levels were assessed using a two-tailed unpaired *t*-test. All data are presented as a mean ± SE. *p*-values < 0.05 were considered statistically significant in all experiments. Statistical significances are represented as **** *p* < 0.0001, *** *p* < 0.001, ** *p* < 0.01, * *p* < 0.1 on respective graphs.

## Figures and Tables

**Table 1:** Top differentially expressed genes.

Name	Gene Name	Fold Change	*p*-Value
*KCNK3*	potassium two pore domain channel subfamily K member 3	−10.0962	2.14 × 10^−47^
*ADGRF1*	adhesion G protein-coupled receptor F1	−7.6674	0.000401
*ASB2*	ankyrin repeat and SOCS box containing 2	−6.47163	5.87 × 10^−7^
*CA9*	carbonic anhydrase 9	−6.24692	4.52 × 10^−11^
*SERPINB2*	serpin family B member 2	−5.76461	1.43 × 10^−14^
*LIMS3*	LIM zinc finger domain containing 3	−5.46518	0.000532
*IGFBP3*	insulin like growth factor binding protein 3	−5.19763	1.84 × 10^−92^
*CXCR4*	C-X-C motif chemokine receptor 4	−5.06219	0.001505
*H4C11*	H4 clustered histone 11	−4.48386	8.04 × 10^−5^
*F3*	coagulation factor III, tissue factor	−3.49677	2 × 10^−99^
*KCNJ16*	potassium inwardly rectifying channel subfamily J member 16	7.475734	0.000122
*CXCL10*	C-X-C motif chemokine ligand 10	6.478581	2.44 × 10^−11^
*SLC28A3*	solute carrier family 28 member 3	6.01688	1.94 × 10^−8^
*VCAM1*	vascular cell adhesion molecule 1	5.927966	0.000336
*TNFSF15*	TNF superfamily member 15	3.688883	1.11 × 10^−27^
*CXCL11*	C-X-C motif chemokine ligand 11	3.627269	2.13 × 10^−6^
*C2CD4A*	C2 calcium dependent domain containing 4A	3.020804	2.63 × 10^−5^
*LRRC26*	leucine rich repeat containing 26	2.995989	5.78 × 10^−8^
*CFB*	complement factor B	2.855559	1.5 × 10^−12^
*LRG1*	leucine rich alpha-2-glycoprotein 1	2.847027	0.000764

## Data Availability

The RNA-seq data generated in the study have been deposited in NCBI Gene Expression Omnibus with the accession number GSE252406.

## Supplementary Materials

The following supporting information can be downloaded at: https://www.mdpi.com/article/10.3390/ijms25031783/s1.
