# Supplementary material for: Transcriptome Analysis Reveals Anti-Cancer Effects of Isorhapontigenin (ISO) on Highly Invasive Human T24 Bladder Cancer Cells
Source: Int J Mol Sci. 2024 Feb 1;25(3):1783. doi: 10.3390/ijms25031783 (PMC10855786; doi:10.3390/ijms25031783)
Supplement: Supplementary file 1 [file ijms-25-01783-s001.zip › Supplemental Figure S1.pdf]

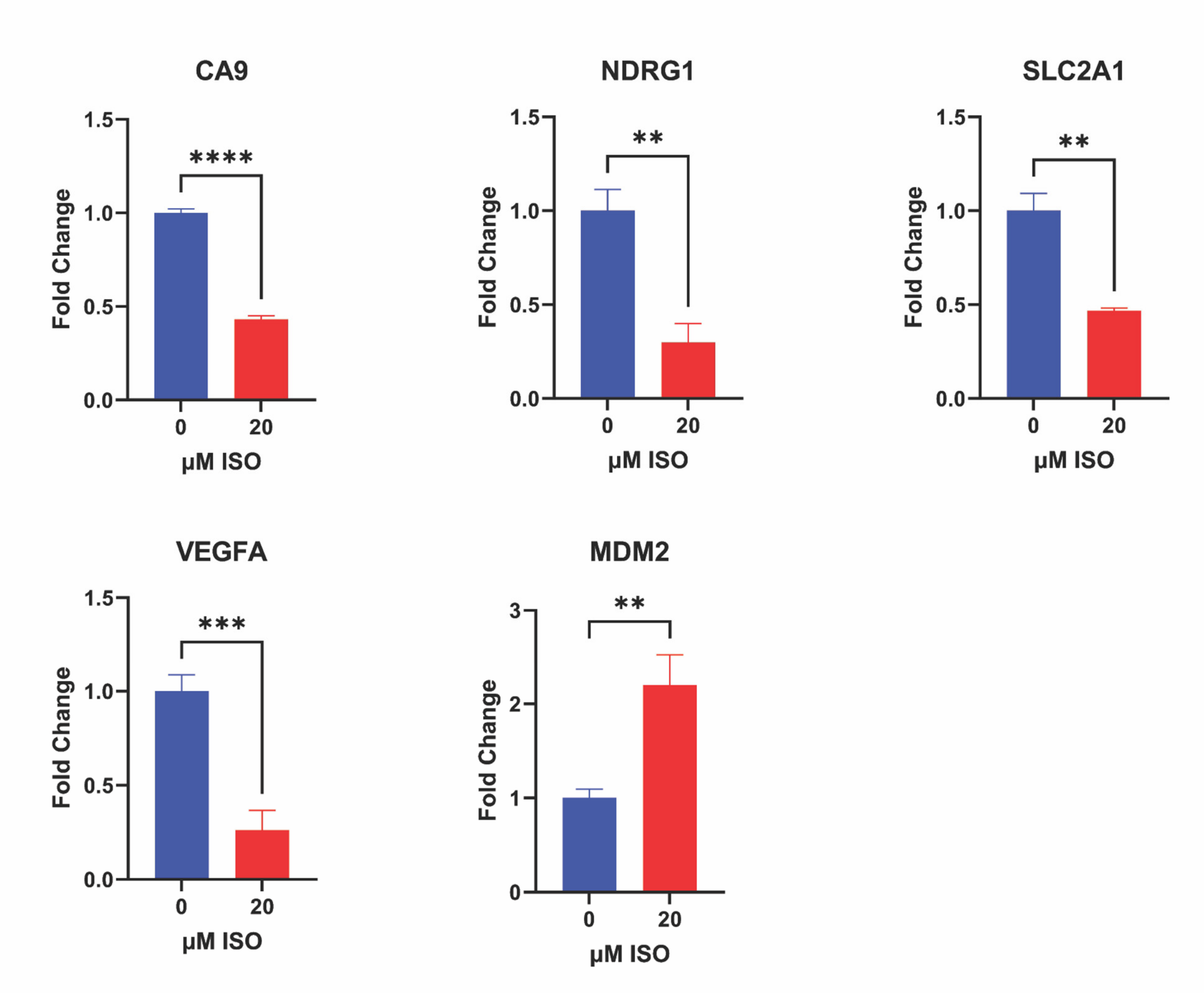

Supplemental Figure S1. Validation of gene expression in 5637 cells by quantitative real-time PCR. Relative mRNA expression levels of *CA9*, *NDRG1*, *SLC2A1*, *VEGFA*, *and MDM2* were analyzed in 5637 cells treated with ISO for 24 h or untreated control cells. Gene expression was normalized relative to the expression of (β-Actin, and presented as fold change to the levels of untreated control cells. Data are presented as mean ± SD (n=3). Significance was determined using unpaired t-test. *P < 0.05 **P < 0.01 ***P < 0.001.
