# Supplementary material for: Transcriptome Analysis Reveals Anti-Cancer Effects of Isorhapontigenin (ISO) on Highly Invasive Human T24 Bladder Cancer Cells
Source: Int J Mol Sci. 2024 Feb 1;25(3):1783. doi: 10.3390/ijms25031783 (PMC10855786; doi:10.3390/ijms25031783)
Supplement: Supplementary file 1 [file ijms-25-01783-s001.zip › Supplemental Table S2.pdf]

Supplemental Table S2: Primers used for qPCR

Gene	Forward Primer (5'-3')	Reverse Primer (5'-3')
CA9	CATCCTAGCCCTGGTTTTTGG	CCTTCTGTGCTGCCTTCTCAT
ITGB4	GCTTCACACCTATTTCCCTGTC	GACCCAGTCCTCGTCTTCTG
NDRG1	AATGCAGAGTAACGTGGAAGT	TGGTCGCTCAATCTCCAGGTC
SLC2A1	TCACTGTCGTGTCGCTGTTT	ACGATGAACCATGGGATGGG
VEGFA	GGAGGCGCAGCGGTTTAG	AACCCGGATCAATGAATATCAA
ACTB	GGACTTCGAGCAAGAGATGG	CCACGTCACACTTCATGATGG
